# Noncontact Body Temperature Measurement: Uncertainty Evaluation and Screening Decision Rule to Prevent the Spread of COVID-19

**DOI:** 10.3390/s21020346

**Published:** 2021-01-06

**Authors:** Giovanni Battista Dell’Isola, Elena Cosentini, Laura Canale, Giorgio Ficco, Marco Dell’Isola

**Affiliations:** 1 Pediatric Clinic, Department of Surgical and Biomedical Sciences, University of Perugia, 06121 Perugia, Italy; giovannibattista.dellisola@studenti.unipg.it; 2 Internal Medicine, Angiology and Atherosclerosis, Department of Clinical and Experimental Medicine, University of Perugia, 06121 Perugia, Italy; elena.cosentini@studenti.unipg.it; 3 Department of Engineering, University of Naples “Parthenope”, 80143 Naples, Italy; laura.canale@uniparthenope.it; 4 Department of Civil and Mechanical Engineering, University of Cassino and Southern Lazio, 03043 Cassino, Italy; ficco@unicas.it

**Keywords:** body temperature, skin temperature, infrared thermometer, clinical thermometer, skin emissivity, COVID-19, uncertainty, screening protocol

## Abstract

The need to measure body temperature contactless and quickly during the COVID-19 pandemic emergency has led to the widespread use of infrared thermometers, thermal imaging cameras and thermal scanners as an alternative to the traditional contact clinical thermometers. However, limits and issues of noncontact temperature measurement devices are not well known and technical–scientific literature itself sometimes provides conflicting reference values on the body and skin temperature of healthy subjects. To limit the risk of contagion, national authorities have set the obligation to measure body temperature of workers at the entrance to the workplace. In this paper, the authors analyze noncontact body temperature measurement issues from both clinical and metrological points of view with the aim to (i) improve body temperature measurements accuracy; (ii) estimate the uncertainty of body temperature measurement on the field; (iii) propose a screening decision rule for the prevention of the spread of COVID-19. The approach adopted in this paper takes into account both the traditional instrumental uncertainty sources and clinical–medical ones related to the subjectivity of the measurand. A proper screening protocol for body temperature measurement considering the role of uncertainty is essential to correctly choose the threshold temperature value and measurement method to access critical places during COVID-19 pandemic emergency.

## 1. Introduction

The recent spread of infectious diseases such as Severe Acute Respiratory Syndrome (SARS), Ebola and swine influenza, as well as the COVID-19 pandemic, has accelerated the need to reliably and quickly identify potentially infected and contagious people. Such viruses are in fact highly contagious, and there is evidence that they rapidly spread from person to person also through respiratory transmission. Among the ascertained symptoms related to SARS-Cov-2 infection, there is the alteration of body temperature [1]. Therefore, all workplaces and public offices as well as crowded places (e.g., commercial malls, airports and train stations, public transport, gyms, churches, hospitals etc.) should provide a body temperature screening procedure aimed at preventing the access of people with febrile symptoms (e.g., body temperature higher than 37.5 °C).

Temperature screening is therefore proposed as a prerequisite for accessing to controlled areas and facilities. In the United States, in Europe and in most of the countries that are gradually setting restrictions to face the COVID-19 pandemic, temperature controls are becoming a daily ritual [2]. Dealing with the global health emergency due to COVID-19, the World Health Organization (WHO) has promoted the use of thermal imaging cameras for body temperature screening. In fact, in Italy, the Prime Ministerial Decree of 26 April 2020 [3] set the obligation to measure the body temperature of workers and public employees at the entrance to their workplace.

However, measuring body temperature is a complex task, especially when this measurement is aimed at identifying, in a quick and reliable way, infected subjects who can potentially infect others with the SARS-Cov-2 virus. As a consequence, body temperature measurement should be cheap, simple, noninvasive, quick and safe for the operators assigned to the measurement and, on the other hand, sufficiently accurate, reliable and reproducible for the related social and health implications.

Several types of thermometers for body temperature measurement are currently available on the market, each showing specific peculiarities and precautions of use. Traditional clinical thermometers present high reliability, but reading is not always easy and need very long response times. Conversely, electrical clinical thermometers, equipped with a digital display for simple and rapid reading, show a shorter response time compared to traditional thermometers, but still require contact with the probe, and, therefore, they can act as vehicle of transmission of the virus [4]. Only infrared thermometers and thermal imaging cameras allow almost instantaneous and contactless temperature measurements, but they measure skin temperature [5].

Probably the most debated issue in remote temperature measurement techniques is its reliability. In fact, such measurement is particularly influenced by the unavoidable instrumental uncertainties and by the operator’s ability, but also by numerous “influence quantities”, such as (i) the emissivity and the reflection coefficient of the emitting skin surface [6]; (ii) the transmission coefficient of the medium between the sensor and the target; (iii) the average radiant temperature of the measurement environment (i.e., the reflected temperature); (iv) the distance and consequent size of the target (effect of the size of the source) [7,8]. However, the accuracy of noncontact temperature measurement can be improved by utilizing dual-band or multiband infrared sensing [9,10]. In fact, these sensors, although more costly and complicated, provide compensation of unknown emissivity and of some background noise, since the infrared emitted from the target at different wavelength bands is detected.

The reliability of the body temperature measurement, however, is not only related to metrological issues, but also to the intrinsic complexity and variability of the “subjective” measurand and to the homeostatic mechanisms of body thermoregulation [11,12]. This process is under hypothalamic control, and it is conditioned by several individual factors (e.g., comorbidities, age, physical activity, digestion, stress, use of drugs and smoking), temporal variables (e.g., circadian rhythm, menstrual cycle), spatial variables (e.g., body and skin) and environmental conditions (e.g., indoor/outdoor) [13,14]. In measuring body temperature, it is worthy to distinguish the core temperature from the peripheral one. The first refers to the temperatures of the abdominal, thoracic and cranial cavities, whereas peripheral temperature refers to the skin or subcutaneous tissue and muscles. There is no gold standard in the measurement of core temperature, which instead is estimated by the measurement of other body sites [11,15]. Thus, the measurement accuracy and reliability depend not only on the type of measurement instrument, but also on the areas of the body where the measurement is performed. The areas of the body most commonly used to measure body temperature are the rectum, armpits and oral cavity, while infrared measurements in the tympanic cavity and in other skin areas (e.g., the forehead, temples and neck) are still matter of discussion in the literature [16,17]. Therefore, in body temperature measurement, it is necessary to take into account adequately several inter- and intra-individual variables, environmental and procedural influences affecting the measurement, that can lead to potential “prescreening errors”.

Regardless of the measurement technique, the instrumental uncertainty, the measuring process and the measurand (i.e., skin or core body temperature), national laws and screening protocols generally set a fixed threshold value for the body temperature (typically 37.5 °C). This generic indication, combined with the lack of knowledge of measurement problems, often leads to unconscious and erroneous assumptions. Indeed, a false negative result (i.e., temperature below the threshold) can be found when (i) the subject’s “core” temperature is higher than “skin” temperature; (ii) the mean radiant temperature, skin emissivity or other measurement variables produce negative systematic errors; (iii) individual and environmental variables reduce body temperature. For these reasons, effective decision rules should rely on an appropriate consideration of the related measurement uncertainty role [18].

In this paper, the authors discuss the noncontact body temperature techniques in relation to the individual, temporal, spatial and environmental influence variables. Moreover, a deepen clinical and metrological analysis is carried out with the aim to evaluate the several error causes (due to the measurand, instrument, environment and operator) and to estimate the measurement uncertainty according to ISO GUM [19] and ISO/TR 13154 [20]. Finally, a temperature screening protocol and related decision rule applicable at different operational conditions (e.g., indoor and outdoor) are proposed, taking into account the related measurement uncertainty.

## 2. Materials and Methods

The measuring principle of noncontact IR thermometers and thermal cameras is based on the Stefan–Boltzmann law which relates the maximum quantity of energy (emitted by a “black” body) with the fourth power of the thermodynamic temperature [21]. However, most remote thermometers are sensitive only on a reduced portion of the electromagnetic spectrum, generally in the medium infrared range (λ ranging from 3 to 5 µm) or more frequently in the far one (λ ranging from 6 to 14 µm). Therefore, the measurement is strictly based on Planck’s law, and the thermometer is designed to operate around the peak of maximum emission (at ambient temperature this peak is about 10 µm). Infrared thermometers generally rely on a microbolometric sensor or a thermopile (which are theoretically sensitive over the entire spectrum range). Lenses and filters interposed between the sensitive element and the measurement target modify the spectral range of the sensor, leading the thermometer to reduce the influence of external influence factors, thus measuring almost all of the radiation coming only from the target. The thermometer optics is therefore an integral part of the instrument itself since it does not only focus on the target, but also performs the spectral cutoff of the transmitted electromagnetic radiation. For this reason, abrasive, oily or solvent products can damage or dull it, making the measurement completely unreliable.

Thermal imaging cameras and more complex thermal scanners are also based on the same measurement principle of infrared thermometers, but they are made of (i) a matrix of sensors as sensitive elements; (ii) an optics similar to that of a video camera; (iii) a postprocessing software capable of returning a thermal image colormap (of the entire body or of one element). In these instruments, the infrared radiation is projected onto a matrix of sensors, and each single pixel of the returned image corresponds to a temperature measurement. Therefore, starting from the measured radiation, surface temperature colormaps are obtained, in which each color corresponds to a thermal intensity. In any case, it is possible to quantitatively evaluate the temperature in each single pixel of the image (also by correcting the emissivity, humidity and ambient or reflected temperature) with the assistance of postprocessing software. Some thermal imaging cameras incorporate many features to facilitate the use of the instrument in industrial and civil applications, such as ability to store text comments, voice comments and photographs in the visible spectrum. Of course, for measuring body temperature, the most reliable thermal imaging cameras are those with higher temperature resolution, which is directly related to the sensors’ sensitivity characterized by the signal-to-noise ratio or the noise equivalent temperature difference (NEDT). The advantages of measuring on the entire face and not on a specific and limited spot are that they allow more reliable measurements and less dependence on emissive singularities and temperature nonuniformity of some parts of the face.

Ultimately, when comparing thermal imaging cameras and thermal scanners with infrared thermometers, even with the same accuracy of the sensitive elements, the former can operate without the direct intervention of an operator and from greater distances (up to 3 m) due to the presence of sophisticated optics, and they can evaluate the entire visible spectrum of the measurand with higher accuracy. In addition to normal thermal imaging cameras, thermal scanners perform a scan of the entire environment, thus allowing the measurement of the temperature on multiple subjects at the same time. The more sophisticated models show also recognition functions to check the use of the mask and to open automated turnstiles after a positive check.

**Figure 1:**
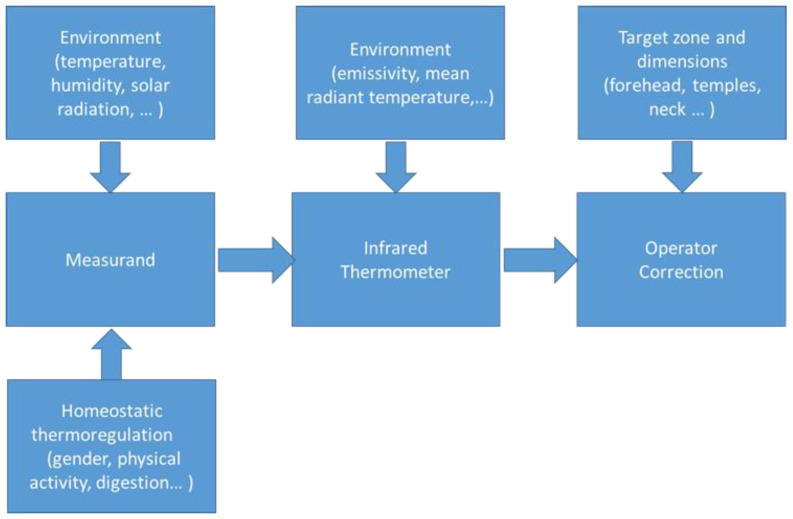
Measuring chain of noncontact body temperature measurement.

**Figure 2:**
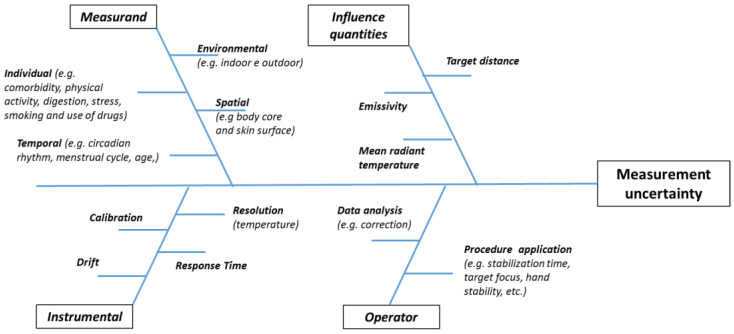
Root causes of noncontact temperature measurement uncertainty.

Accuracy and reliability of remote body temperature measurement depend on multiple effects, such as (see Figure 1) (i) the intrinsic variability of the measurand (due to both environment factors and individual homeostatic thermoregulation); (ii) the measurement procedure used (due to both instrument accuracy and environmental factors); (iii) the data processing by the operator (in terms of understanding and consequent data corrections). Therefore, as shown in Figure 2, the causes of uncertainty depend on measurand (i.e., individual, spatial, temporal and environmental factors) instrument (e.g., temperature resolution, accuracy and drift), effects of influence quantities (e.g., emissivity and mean radiant temperature) and operator (e.g., operator ability, instrument setting and data postprocessing).

### 2.1. Measurand Uncertainty

#### 2.1.1. Individual and Spatial Parameters

The first issue in analyzing body temperature measurement and evaluating intra- and inter-individual variability is to define the average temperature of the healthy population. Wunderlich [22] was among the first in 1868 to evaluate the body temperature in a large sample of the population, and he established the axillary mean value of 37 °C within a range of 36.2 to 37.5 °C. Subsequent studies questioned Wunderlich’s result [23] highlighting the poor reliability of the axillary measurement and the uncertain accuracy of the thermometers of the time.

However, considering the numerous physiological variables influencing the body temperature, it is not possible to define a specific “normal” temperature value, but rather a range of normality between 36.5 and 37.5 °C [13]. The thermoregulation system is under hypothalamic control, but, as mentioned above, body temperature can significatively vary from core to peripheral zone. Core temperature is almost homogeneous, and within certain limits, it is not influenced by environmental effects. On the contrary, the peripheral temperature varies between the body areas where it is measured, based on variables including the subcutaneous adipose layer [24,25], local blood flow, metabolic activity, environmental conditions and sweating [26,27]. In fact, the skin was described by Henane as a thermal mosaic [28]. Burton [29] proposes the calculation of the mean body temperature (MBT) based on the principle that the temperature of the internal “body core” tissues is almost homogeneous. The MBT is a weighted average generally estimated using the Equation (1):

(1) $$MBT\;=\;0.4\;T_{skin}\;+\;0.6\;T_{r}$$

in which $T_{r}$ is the rectal temperature and $T_{skin}$ is the skin temperature estimated as a weighted average of the temperatures measured in different areas of the body.

**Figure 3:**
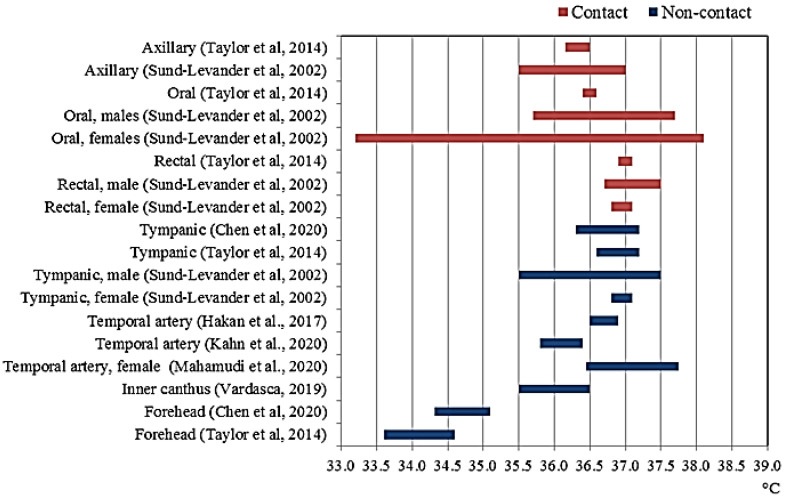
Measured body temperature variability at different body sites.

Certainly, the sites of the body most commonly involved in measuring body temperature are the axilla, the oral cavity and the rectum. Among these, the rectal temperature has long been considered the most reliable surrogates of the core temperature for its thermal stability and high mean values [30]. However, measurements in the tympanic cavity area and other parts of the skin such as the forehead, the temple (i.e., temporal artery) and face (i.e., inner canthus) are becoming increasingly common, especially in noncontact temperature screening. In hospitals, other body areas are theoretically suitable for body temperature measurements, such as the bladder, esophagus, pulmonary artery. Taylor [15] and Sund-Levander [31] conducted a systematic study of the temperature variations between different areas of the body. In Table 1 and Figure 3, the temperature variations measured in different body sites in healthy population are reported, together with the main related advantages and disadvantages.

The clinical relevance of body temperature measurement derives from the capacity of various pathological conditions to alter the thermoregulation mechanisms through endogenous and exogenous pyrogens. Infectious diseases are the typical example associated with important deviations from the basal values of body temperature, sometimes reaching values of hyperpyrexia. However, there are other comorbidities that can often cause mild changes in body temperature that may go unnoticed, such as hypothyroidism (associated with average temperature reductions) and neoplastic pathologies (associated with temperature increases) [36]. Psychiatric disturbances can also lead to changes in temperature as demonstrated by Nikitopoulou et al. [37] and Rausch et al. [38]; according to their studies, patients with depression have body temperature values increased by 0.1 and 0.25 °C, respectively, compared to normal.

Age represents an additional variable to consider in controlling body temperature. Elderly people, due to a reduced physical activity and a lower efficiency of the thermoregulation mechanisms, maintain lower average temperatures [39]. In particular, rectal, ear, oral and axillary temperatures are 0.2–0.7 °C lower than the corresponding average reference temperatures [40].

It has also been demonstrated that some drugs can cause a change in body temperature. In particular, oral contraceptives (synthetic steroids) can lead to a persistent increase in body temperature of about 0.6 °C compared to that of women who do not use them. Therefore, the body temperature appears to be comparable to that of women in the luteal phase, maintaining the normal cyclicality, with a preovulatory nadir [41]. Furthermore, since exogenous steroids have a greater power than endogenous, their effect can persist even after a long period. Exogenous administration of melatonin can also induce a hypothermic effect with a logarithmic dose-response effect. Doses of 5 mg of melatonin induce a lowering of the internal temperature of about 0.2 °C; higher doses, on the other hand, do not induce a further substantial reduction in body temperature [42]. Even opiates, anti-H1 and anti-H2 antihistamines can cause a change in temperature, which depends on the dosage and the route of administration. Oral administration of diphenhydramine can induce a reduction of 0.6 °C on average as well as 30 mg of subcutaneous morphine [43].

One of the main factors related to body thermoregulation is the percentage of subcutaneous fat, which, as a thermal insulator, alters heat dissipation. It is therefore easy to understand how obese people tend to cool down more slowly when passing from hot to cold environments and, at the same time, tend to be more at risk of hot thermal stress [44,45]. However, there is no proven correlation between obesity and body temperature, and although some [46,47] support an inverse association between temperature and obesity, others [48] deny this hypothesis.

In conclusion, despite the individual variables can cause significant changes in basal temperature, it is difficult to take them into account in the instrumental temperature screening due to the impossibility of identifying a priori individuals suffering from comorbidities or who have taken drugs. A separate discussion could be made on the age of individuals.

#### 2.1.2. Temporal Parameters

**Figure 4:**
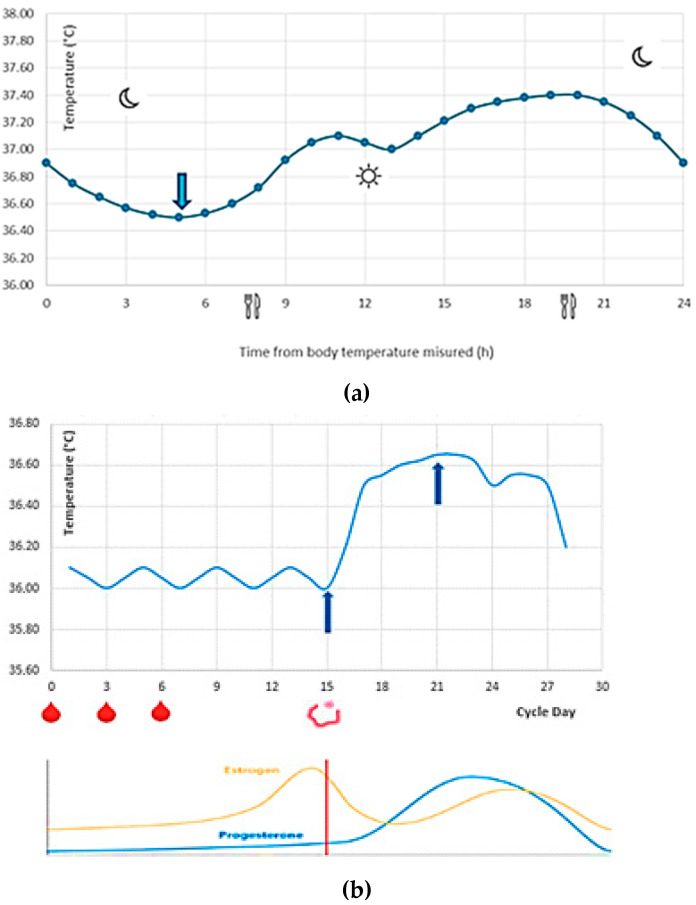

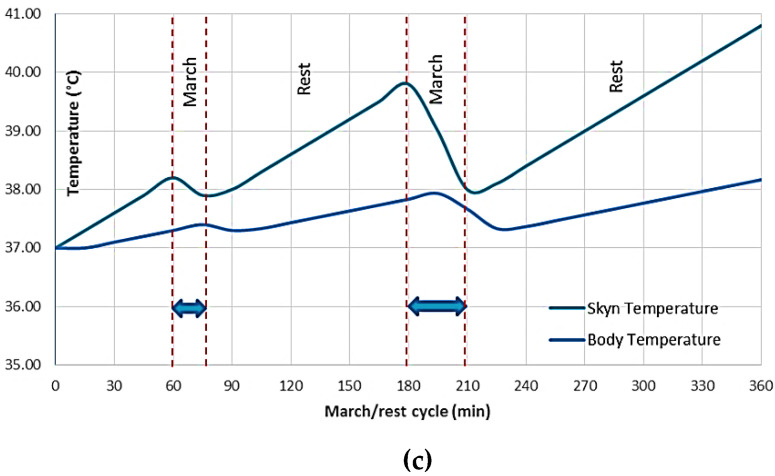
Typical body temperature trend: (**a**) circadian rhythm (the blue arrows represent respectively the minimum and the maximum peak during the day); (**b**) ovulatory cycle and hormonal concentration (the blue arrows represent the minimum and the maximum peak during the month, and the red drops represent the menstruation); (**c**) march/rest cycle (the metabolism due to muscle activity, in particular the blue arrows represent the march cycle and the remaining one the rest cycle)**.**

In order to optimize physiological processes, body temperature follows a circadian rhythm (Figure 4a), mediated by endogenous and exogenous factors, which in a conventional lifestyle provides a “plateau” from 14:00 to 20:00 and a minimum peak at 5:00 [49]. In particular, in natural conditions of lighting and social interaction and setting the sleep duration from 23.00 to 7:00, body temperature ranges from 36.5 °C at 4 a.m. to 37.4 °C at approximately 8:00 p.m. [50].

A body temperature variability was also found after meals, but it was not significant. In fact, it has been demonstrated, through the use of an ingestible capsule with a telemetry sensor, that there are no significant alterations in body temperature after a light mixed meal of about 600 kcal (premeal temperature of 37.3 ± 0.3 °C and postmeal temperature of 37.2 ± 0.23 °C) [51]. These data have been confirmed by Hoffmann et al. [52] with a study considering three meals with the following macronutrient composition: 5% protein, 35% fat and 50% carbohydrates.

The cyclical hormonal changes related to the menstrual cycle lead to a monthly variability of the body temperature in the females with higher temperatures in the luteal phase (36.5–36.8 °C), during which there is an increase in progesterone, and lower temperatures in the preovulatory phase (35.9–36.4 °C), during which there is an increase in estrogen [53], as depicted in Figure 4b.

When the endogenous heat production exceeds the body’s capacity for dispersion (e.g., during physical activity), an increase in skin and body temperature occurs (Figure 4c). In extreme cases, the increase in metabolism due to intense muscle activity leads to the so-called exercise-induced hyperthermia (i.e., an increase in the rectal temperature up to 40 °C) [54]. Unlike parainfectious fever, in which the hypothalamic temperature target resets to higher values, during hyperthermia, the cessation of the heat source determines a rapid return to basal temperature values (within 30 min) [55]. Therefore, before performing a body temperature measurement, it would be advisable to wait a reasonable time after some activities (e.g., intense physical exercises, hot baths, intake of hot/cold food and drinks, etc.). Only in this way the temporal variability of the body temperature can be considered negligible.

#### 2.1.3. Environmental Parameters

In a moderate environment, the peripheral temperature can be 2–6 °C lower than the core one, although this gradient can range from almost zero (in hot environments) to high values (in cold environments). In [56], average temperatures measured at nine different body areas at different room temperatures are reported. The authors found large difference between core and peripheral temperature (up to 10–15 °C for foot and finger) in a warm environment (about 20 °C), whereas these differences were lower (about 1–2 °C) in a hot environment (about 33 °C).

Skin temperature measurement is commonly used to explore the interaction between human thermophysiology and the external environment [57]. While the core temperature is endothermic and strictly regulated by the brain, the skin temperature is exothermic since it is influenced by the environment and by the “dual-thermic” thermoregulation ability. Particularly during heat stress, peripheral vasodilation increases the blood flow of the skin (with consequent increase in temperature and heat dissipation). On the other hand, during cold stress, peripheral vasoconstriction leads to a decrease in skin temperature and heat transmission to the environment.

Therefore, in the case of sudden changes in environmental conditions, it would be advisable to wait an adequate stabilization time to reach a new steady state before the measurement.

### 2.2. Instrumental Uncertainty Causes

The main causes of instrumental uncertainty are (i) uncertainty of the characteristic (calibration curve), (ii) drift and (iii) temperature resolution.

Noncontact infrared thermometers show measuring accuracies that are on average lower than those of contact ones. Clinical infrared (IR) thermometers may be classified into two types: “ear canal IR thermometers” and “skin IR thermometers. ASTM E1965–98:2003, EN 12470-5:2003 and EN ISO 80601-2-56:2012 [58,59,60] provide specifications for infrared thermometers. In particular, maximum permissible errors of the infrared thermometer equal 0.2 °C for the auditory canal and 0.3 °C for the skin have been set at normal range and environmental conditions. On the other hand, according to ASTM E 667-86 and ASTM E 1112-86, the maximum permissible error of mercury in-glass and electronic thermometers is equal to 0.1 °C.

In regard to thermal imaging cameras, ISO IEC 80601-2-59:2011 [61] provides relevant technical and performance elements aimed at performing reliable measurement. In particular, the standard provides (i) threshold temperature (adjustable in the range between 34 and 39 °C, with increments not exceeding 0.1 °C); (ii) minimum display interval for face temperature (between 30 and 40 °C); (iii) temperature resolution better than 0.1 °C; (iv) availability of alarm systems (e.g., above 37.5 °C); (v) image processing software to detect the febrile state of subjects (e.g., individual, row-ordered group or unordered group). In the case of research systems on people groups for a screening, the software must implement the automatic recognition of different subjects (targets). Thus, the accuracy in estimating the temperature depends on the performance of the system components (i.e., the camera and the data postprocessing software), the detection methods and the environmental conditions. The best accuracy (typically within 0.3 °C) is obtained in a controlled environment for individual control, whereas accuracy worsens up to 2 °C for unordered people groups in uncontrolled temperature environments. Finally, it is necessary to carefully follow the indications provided in the technical note ISO/TR 13154:2017 [20] to ensure proper use of these systems and correct interpretation of the results.

The drift of IR thermometers and thermal cameras strongly depends on the quality of the instrument and of the conditions of use, and it is typically about 0.1 °C/year. This uncertainty source can be reduced through frequent periodic calibrations. Infrared blackbody calibrators are typically used for calibration since they rely on a surface which emissivity ranges between 0.95 and 0.98 heated at different known temperatures. The temperature resolution of the measuring instruments depends on the number of bits of the A/D converter and the consequent choice of the LCD display. In the case of infrared thermometers, temperature resolution is generally equal to 0.1 °C.

### 2.3. Environmental Uncertainty Causes (Influence Quantities)

#### 2.3.1. Temperature Effect

Nowadays, the most used infrared cameras and IR thermometers use bolometric/microbolometric technology. The main advantage of bolometric technology when compared to photonic technology is that a cooling system to operate in the long-wave infrared band is not needed. On the other hand, the thermal frames measured by noncooled microbolometric IR cameras are hardly influenced by the spatial nonuniformity noise. Generally, this effect varies with time due to the internal camera temperature stability (i.e., lens, camera surroundings and Focal Plane Array (FPA), and the thermal drift can be particularly relevant. Unfortunately, the ambient temperature fluctuations are unavoidable; thus, compensation of the instrument output through frequent multipoint calibration or using adaptive signal-processing-based algorithm statistics is required [62]. The influence of the device temperature on the temperature measurement strictly depends on the type of sensor, and the related error can be very high; thus, adequate compensation strategies have to be adopted by manufacturers (e.g., chopper) or by final users (e.g., the operator should avoid holding the instrument in their hands or leaving it at low temperatures for long times).

#### 2.3.2. Skin Emissivity

Bodies in nature exhibit very different behavior from black bodies and emit only a reduced amount of energy compared to the maximum one. As a consequence, they reflect a certain amount of the energy incident on the surface itself in a complementary way to that emitted (according to Kirchhoff’s law). For this reason, it is necessary to avoid performing measurements in the presence of direct solar radiation on the measurand surface.

**Figure 5:**
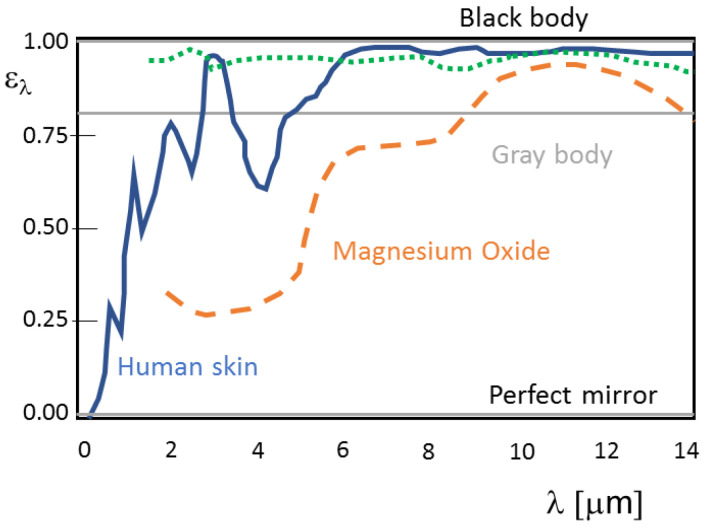
Monochromatic emissivity trend of human skin and other surfaces.

To quantify the effect of the surface emissivity, manufacturers of clinical thermometers directly set the emissivity value ε (i.e., the ratio between the energy emitted by the real body and that of the black body at a given temperature) in the instrument and correct the value of the reflected energy, generally considering this is equal to that produced by an environment at 20 °C. However, the monochromatic emissivity trend of real bodies at a fixed temperature should be very different (see Figure 5) due to the constituting material, the surface finish and the wavelength. In fact, total emissivity is not constant with the temperature surface.

Human skin shows a very high far infrared emissivity (regardless of skin color) of approximately 0.98 [6], but numerous parameters can influence its value (e.g., sweat, make-up, lotions, scars, porosity of the skin, hair, etc.) [63]. The emitting properties of the skin are mainly related to melanin which absorbs in the ultraviolet region and also determines the color of the skin. This latter presents a wide absorption and emission band of around 0.275 µm due to aromatic chromatophores. At higher wavelengths (in the visible and near-infrared), absorption peaks at 0.76, 1.00, 1.20, 1.45 and 2.00 µm (due to the presence of water) are present. In addition, other substances present on the epidermis, including keratin, collagen, fats, melanin, make-up and water, affect the skin’s absorption/emission spectrum.

Skin emissivity depends on its humidity. In fact, water has a far infrared emissivity of 0.96, while that of oils and fats is about 0.82. Therefore, in the infrared range, skin emissivity decreases as humidity increases, and the minimum value corresponds to skin completely covered in sweat. The emissivity of clay and earthy pigments, on the other hand, ranges between 0.93 and 0.95. For this reason, the noncontact temperature measurement should always be carried out in a spot of clean and dry (i.e., sweat-free) skin, possibly avoiding the presence of hair, wounds or scars, cosmetics or other products that could greatly affect the measurement. Fever of nearly 1–2 °C could be masked by applying common cosmetics containing solid particles to the human forehead [64].

**Figure 6:**
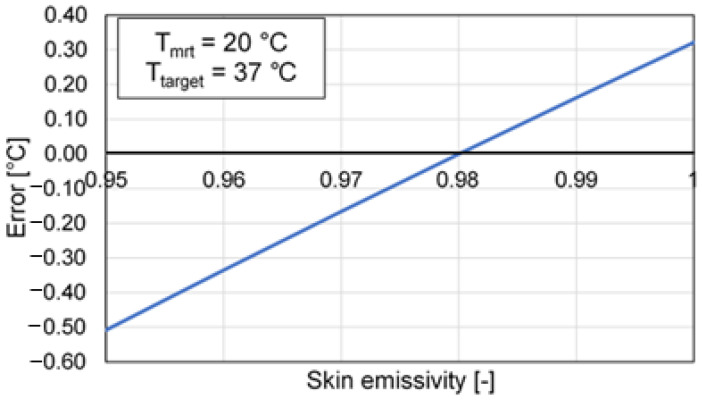
Error trend depending on skin emissivity.

Figure 6 shows the effect of the skin emissivity variation on the measured temperature.

#### 2.3.3. Surrounding Environment (Mean Radiant Temperature) and Other Factors

**Figure 7:**
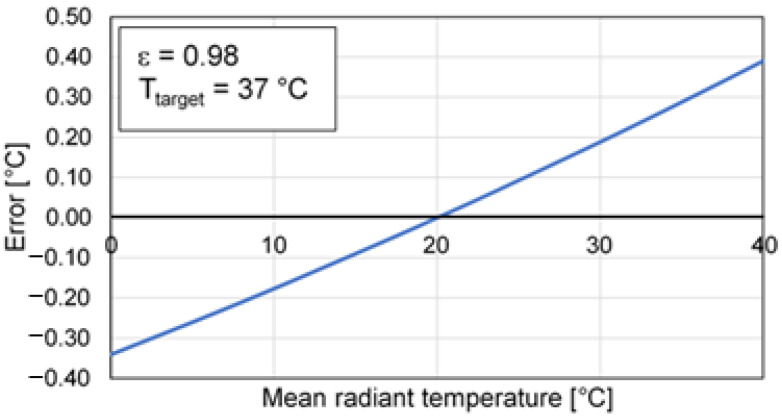
Error depending on the mean radiant temperature.

The radiation of the surrounding environment can determine a significant influence especially when the emissivity of the skin is lower than the reference value (i.e., 0.98), and, therefore, it is no longer possible to neglect the radiation reflected by the target compared to that emitted. As an example, when the emissivity is equal to 0.98 and the mean radiant temperature is 10 °C higher or lower than the body temperature (i.e., outdoor conditions), an error of about 0.2 °C will occur. On the other hand, these errors cannot be corrected through a simple calibration, since this depends on the reflected ambient temperature (e.g., from a window that is irradiated by the sun), even if the room temperature is stable [9,10]. The use of a reference target (i.e., at known temperature) may reduce and compensate for the effect of this influence factor. In this regard, Figure 7 shows the dependence of the error on the mean radiant temperature, having set a calibration temperature equal to 20 °C.

**Figure 8:**
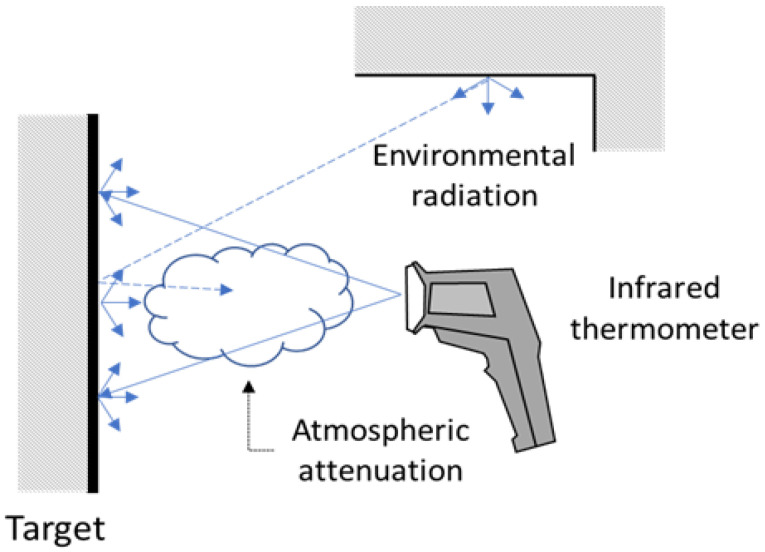
Other influence factors.

Other influencing factors are represented by the attenuation of the radiation due to the atmosphere (this effect is generally negligible when short distances between the instrument and the target are guaranteed) or glass/filters interposed between the measurement target and the instrument (see Figure 8).

### 2.4. Operator (Setting and Data Processing)

#### 2.4.1. Angle of Incidence

**Figure 9:**
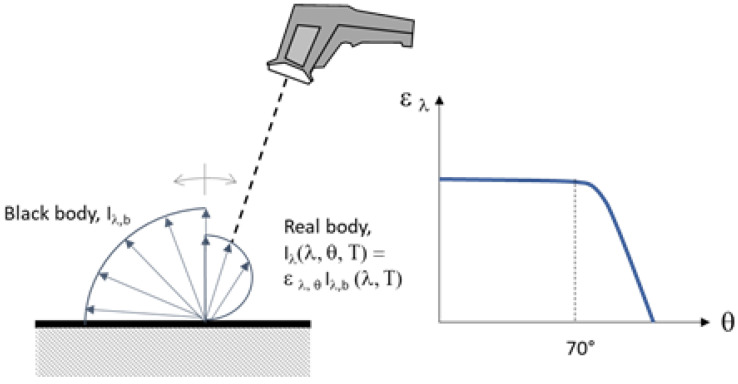
Influence of angle of incidence.

Several factors related to the operator can affect the body temperature measurement accuracy due to both the procedure (e.g., angle of incidence of the measurement and distance from the target) and to the postprocessing of the data. A further complication of the measurement is represented by the fact that the emission angle of the IR radiation for all real bodies is not perfectly Lambertian, such as that of black bodies. Therefore, for accurate measurements, the angle of incidence should be kept lower than 60–70° (see Figure 9).

#### 2.4.2. Target Distance and Focusing

**Figure 10:**
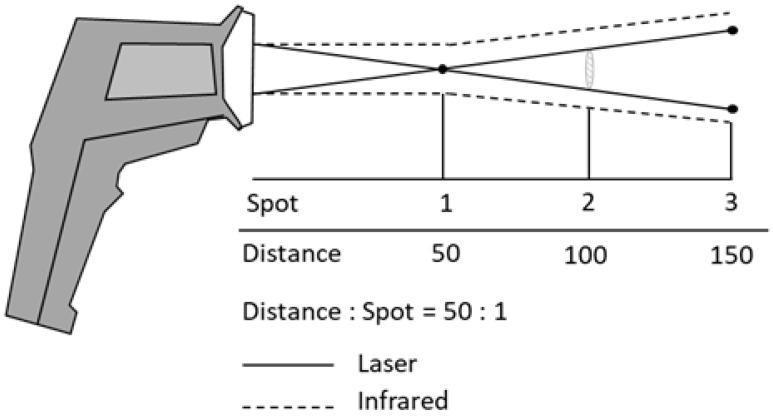
Effect of the target distance (D:S).

A specific measurement issue of infrared thermometers is represented by the target (or spot) dimension, which must coincide with the surface that is actually intended to be measured. In fact, the infrared radiation emitted by the target passes through the thermometer optics, and it is projected into the sensor. If the measured target is smaller than that of the “spot”, the sensor will also be hit by radiation sources coming from the immediate vicinity of the target. In this case, the thermometer does not measure the temperature of the target, but an average of the temperatures of the target and of the surrounding emitted surfaces. The dimensions of the measurement target are, in fact, closely related to the distance of the thermometer from the target itself. The higher this distance, the larger the spot size. Consequently, when small targets are measured, the thermometer should be kept close to the target itself (see Figure 10).

Manufacturers’ technical specifications specify the distance ratio on the spot area (D:S) or the spot size ratio (SSR). For example, the SSR of most infrared thermometers ranges between 1:5 and 1:50, meaning that they can measure the temperature of a 1 cm diameter target at a distance ranging from 5 to 50 cm.

A further crucial factor for thermal camera is target focusing, because images out of focus strongly affect the accuracy of results. Three types of focus system can be chosen in thermal cameras: (i) fixed focus (i.e., focuses on targets at a specific distance) suitable for low-temperature resolution and quickly finding hot and cold spots; (ii) manual focus used to obtain an accurate focus (e.g., very close to the target) for high-temperature resolution measurements; (iii) autofocus: (e.g., laser-assisted, multifocal image capture) used to obtain sharp focusing for both experienced and novice operators.

## 3. Results

### 3.1. The Proposed Screening Protocol

Thermal measurement screening can be useful to separate potentially infectious individuals in accessing workplaces sites or crowded locations, such as hospitals, clinics, critical infrastructures, universities, schools, public offices (e.g., police and fire stations, museums etc.), public transport etc. For example, the appropriateness or mandatory nature of measuring the body temperature of workers and employees at the entrance to their workplaces and offices was set in Italy by the Prime Ministerial Decree of 26 April 2020 [3]. However, the prevailing guidelines suggest measuring the body temperature at the entrance of each work activity according to the precautionary principle.

In theory, two different types of screening could be adopted to control body temperature: (i) the first one, based on a deterministic temperature threshold (generally set at 37.5 °C to avoid a large number of false positives); (ii) the second one, based on a statistical threshold value determined on the basis of the sample-measured temperatures at real measurement conditions and the adopted procedure. In order to be consistent with the decision rules for the selection of suspicious cases, in the first case, it is necessary to take into account both the accidental and systematic uncertainty contributions (i.e., those depending on the design/choice phase of measurement method and on the on-field execution phase). In the second case, if the measurements are always carried out with the same instrument, measurement method, environmental conditions and operator, it is possible to take into account only some uncertainty causes (e.g., only type A uncertainties that do not determine a significant difference between the measurement of a single subject and the mean value of the population under screening). In other words, if the environmental conditions determine an uncertainty of the measurement of 0.3 °C but are stable and produce a systematic measurement shift (that is always constant in excess or in defect), this uncertainty should not be taken into account in the screening procedure. This circumstance can greatly simplify the prescreening phase considering negative subjects which in absolute terms have temperatures compatible with the absolute threshold value, but which do not exceed this value. A second step of the screening projection can therefore concern only the subjects that exceed the absolute limit value with more accurate measurement techniques.

In any case, effective screening cannot ignore the clear definition of the body site where the measurement has to be performed (e.g., the frontal forehead, inner canthus etc.) and may refer to this value as the corresponding core temperature value. The measurement protocols issued by the competent authorities establish that workers should “be subjected to temperature measurement”, and this does not necessarily imply the presence of a measurement operator. In principle, self-measurement by the worker could also be carried out under the control of the employer or his subordinate who should ensure the measurement correctness. The legislator leaves the freedom to choose the most suitable measuring instrument: both innovative remote thermometers and traditional clinical contact thermometers (i.e., analog liquid expansion and digital electric). However, the latter would not guarantee in many situations an adequate measurement time, also due to the need to disinfect the thermometer each time it is used (unless personal probes or disposable strips are used for each worker). It should also be noted that compared to traditional contact thermometers, infrared thermometry allows for the maintenance of a greater distance between the operator and the worker subject to measurement. In particular, infrared thermometers need to measure at about 5–15 cm from the subject (due to the limited SSR), while thermal imaging cameras and thermoscanners can measure at distances of a few meters. These considerations often guide the employer towards the choice of a remote temperature measurement method (i.e., an infrared thermometer, a thermal imager or a thermoscanner).

### 3.2. Uncertainty Estimation

In order to choose the most appropriate thermometer and measurement method, it would be necessary to evaluate its main metrological characteristics (in addition to response time and easiness of use) which influence the measurement uncertainty and reliability. On-field uncertainty estimation should include all relevant components of uncertainty and not only instrumental ones. In fact, the measurement uncertainty strictly depends on the measurand, instrument, test conditions and procedures used by the operator in the execution and data processing [65,66].

Often, noncontact thermometer manufacturers do not report metrological specifications in terms of measurement accuracy, meaning that the display temperature resolution (which is typically equal to 0.1 °C) is consistent with its accuracy. Typically, the expanded uncertainty of body temperature measurement through traditional contact thermometer is within 0.1 °C, whereas that through infrared thermometers can be higher than 0.2 °C. In addition, the measurement procedure (e.g., measurement performed after thermal stabilization of the subject or not), the body site (e.g., the forehead, temple or tympanum), the skin condition (e.g., the presence of make-up or sweat) and the hour of the day (e.g., before or after meals) may considerably increase the uncertainty value.

Table 2 shows an example of uncertainty budget for noncontact body temperature measurement in two different typical conditions: (i) indoor after thermal stabilization of the subject and (ii) outdoor without thermal stabilization of subject. From the calculation of the combined standard uncertainty, it can be observed that the measurement uncertainty can be influenced by the measurement conditions more than by the instrument. Furthermore, the combined uncertainty is almost higher in the case of “uncontrolled” conditions, unless the statistical threshold is considered.

Therefore, according to ISO GUM [19], the combined standard uncertainty is given by the Equation (2), whose terms are described in Table 2.

(2) $$u_{c}^{2}=u_{meas}^{2}+u_{inst}^{2}+u_{env}^{2}+u_{oper}^{2}\;\;=u_{m,\;ind}^{2}+u_{m,temp}^{2}+u_{m,env}^{2}+u_{i,\;cal}^{2}+u_{i,drift}^{2}+u_{i,res}^{2}+u_{e,\;temps}^{2}+u_{e,\;emi}^{2}+u_{e,\;mrt}^{2}+u_{o,\;\;target}^{2}$$

### 3.3. Conformity Decision Rule

**Figure 11:**
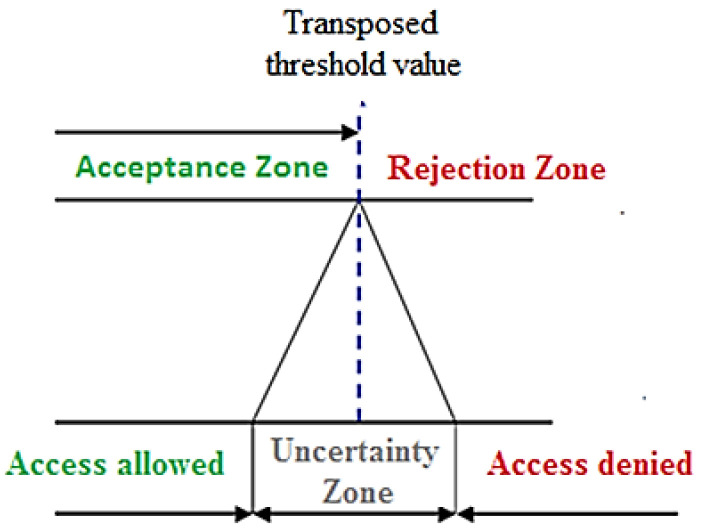
Acceptance, rejection and uncertainty zones.

In order to obtain the appropriate decision rule in assessing conformity to specifications (in our case the temperature measurement above the threshold), the following aspects should be considered:

(a) A threshold reference value should be set by the decision maker (e.g., by law), by declaring at which body site the measurement is to be performed, and this applies when a fixed threshold value is used instead of a statistical one (e.g., 37.5 °C in the case of axillary contact measurement).
(b) For body sites other than that fixed (by law), the threshold value should be adequately transposed; as an example, in the case of noncontact forehead measurement, the threshold limit should be transposed by about 1.6 °C as a function of the most reliable literature reference value [16] and then set to 35.9 °C. This value has been considered sufficiently reliable (as shown in Table 1), since the related uncertainty is lower than the individual, spatial and temporal ones, as shown in the uncertainty budget in Table 2. In any case, when the transposition associated with the measurement technique used is not supported by extensive literature studies, a further uncertainty on this correction should be considered.
(c) Finally, a corrected conservative threshold value should be considered with the aim of properly taking into account the unavoidable measurement uncertainty, meaning that this latter (e.g., 0.4 °C for indoor conditions) should determine an uncertainty zone centered on the transposed threshold value as depicted in Figure 11.

Therefore, the measurement uncertainty should be carefully estimated and possibly reduced to avoid treating subjects with slightly altered body temperature as a “false negative” and to reduce the number of “false positive”. In fact, the greater the uncertainty of measurement, the greater the number of false positives that require treatment after the prescreening phase.

As above described and following a precautionary principle, the authors suggest adopting a double-step measurement procedure. In the first step, a simple and quick (although less accurate) noncontact temperature measurement is performed to assess whether the temperature of the subject is below the above-defined transposed threshold limit (e.g., 35.9 °C), further decreased by the measurement uncertainty (e.g., 0.4 °C which is typical of an infrared thermometer measuring the body temperature in the forehead in an indoor environment).

Only when the first-step noncontact measurement falls within the uncertainty zone (e.g., from 35.5 to 36.3 °C for forehead measurement) is a second step then performed by means of a contact temperature measurement and after the subject has been at rest to thermally stabilize for at least 15 min in an indoor environment. For example, in the case of a second-step axillary temperature assessment with a threshold value of 37.5 °C, a measurement uncertainty of 0.2 °C (i.e., the typical uncertainty of a Galinstan thermometer for axillary body temperature measurement in controlled conditions) should be considered (see Table 3), thus leading to a corrected threshold value equal to 37.3 °C.

Moreover, the threshold temperature values should be evaluated on the basis of real working conditions, and the protocol described should be subject to further refinement based on data collected in a practical environment.

## 4. Conclusions

The numerous measurement methods and devices currently available, together with the related metrological and clinical issues, make the choice of body temperature screening protocols to prevent the spread of COVID-19 particularly complex. To this aim, noncontact temperature measurement method has been identified as the most practical solution considering the short response times, the intrinsic simplicity and the safety for the operators. In particular, thermal imaging cameras and automated thermal scanners can completely avoid the exposure of the operator to the potential risk of contagion and generally show good accuracy due to the possibility of carrying out more complex facial thermal mapping. The high cost of these instruments has led to the spread of infrared thermometers.

However, the reliability of the body temperature measurement depends on different factors, such as (a) the measuring instrument; (b) the body site; (c) the procedure; (d) the environmental conditions; (e) the measurand conditions. In fact, the expanded uncertainty of noncontact body temperature measurement can be greatly higher than the sole instrumental one. For example, the authors estimated expanded uncertainty to range between 0.40 °C (for a subject at rest in an indoor environment and after an adequate stabilization time) and 0.62 °C (for a subject immediately after marching in an outdoor environment and without thermal stabilization). Conversely, expanded uncertainty of contact body temperature measurement in controlled conditions was estimated equal to 0.20 °C.

Therefore, in order to improve the reliability of screening temperature protocols to prevent the spread of COVID-19 disease, according to the precautionary principle, the authors propose the following:

- To set a threshold reference (by considering an assigned measurement body site);
- To punctually establish the measurement conditions and method;
- To accurately estimate the measurement uncertainty (taking into account the main contributions at the real operative measurement conditions);
- To transpose the threshold reference value as a function of the body site used;
- To perform a double-step measurement protocol consisting of (a) a first step, with a noncontact body temperature measurement, and (b) a second step, with a further contact body temperature measurement when the measured value falls within the uncertainty zone.

The application of the proposed protocol reduces false negatives and, as a consequence, also the risk associated with unreliable screening. To further reduce the occurrence also of false positives, particular attention should be paid to (i) the choice of the measuring body site with higher sensitivity and selectivity; (ii) the acclimatization of the subject; (iii) the choice of the thermometer with a higher sensitivity, repeatability and stability; (iv) the frequent calibration of the instrument; (v) the use of a reference target (i.e., at know temperature); (vi) the adequate training of the measuring operator. In this sense, improving a single influence factor, such as the sole sensor sensitivity, does not does lead to significant improvements.

The adoption of the proposed protocol will allow for the combination of the easiness of use and the hygiene of noncontact thermometers with the precision and reliability of contact ones, enabling the reduction of the false negatives due to measurement uncertainty.

## Figures and Tables

**Table 1:** Body temperature measurement site comparison.

Type	Body Site	Mean Reference Temperature in Healthy Subject	Advantages	Disadvantages
contact	Axillary	36.3 °C (36.15–36.5) [15]	Simplicity of use Widespread and well known Noninvasive measurement	Need to lock the patient’s arm Less accurate than tympanic and rectal measurement Cleaning of the thermometer
		36.3 °C (35.5–37.0) [31]		
	Oral (sublingual)	36.5 °C (36.4–36.6) [15]	Simplicity of use Noninvasive measurement	Need to keep mouth closed and to measure away from meals Evaporative cooling during breathing Less accurate compared to rectal measurement Cleaning of the thermometer
		Males: 36.7 °C (35.7–37.7) Females: 36.2 °C (33.2–38.1) [31]		
	Rectal	37 °C (36.9–37.1) [15]	High reliability High accuracy	Unpleasant for subjects Difficulty of positioning Risk of injuries Invasive measurement Need for disinfection
		Males: 37.0 °C (36.7–37.5) Females: 37.0 °C (36.8–37.1) [31]		
noncontact	Tympanic	(36.9 ± 0.3) °C [16]	Simplicity of use High reliable (in absence of ear wax) High accuracy Noninvasive measurement	Risk of injuries Need of disinfection
		36.85 °C (36.6–37.2) [15]		
		Males: 36.5 °C (35.5–37.5) Females: 37.0 °C (36.8–37.1) [31]		
	Temporal Artery	(36.1 ± 0.3) °C [32]	Hygienic Noninvasive measurement	Low accuracy Need to locate the point of max temperature
		(37.1 °C ± 0.65) °C [33]		
		Females: (36.7±0.2) °C [34]		
	Inner canthus (Max facial)	(36.0 ± 0.5) °C [35]	Hygienic Noninvasive measurement No risk of injuries	Low accuracy Need to locate the point of max temperature
	Forehead	(34.71 ± 0.392) °C [16]	Simplicity of use Hygienic Fast measurement Noninvasive measurement No risk of injuries	Less accurate compared to rectal and tympanic measurement
		34.1 °C (33.6–34.6) [15]		

**Table 2:** Uncertainty budget.

Uncertainty Source	Uncertainty Cause	Symbol	Type	Distribution	Measurement Conditions			
					Indoor (After Subject Acclimatisation)		Outdoor (Without Subject Acclimatisation)	
					Expanded Uncertainty	Standard Uncertainty	Expanded Uncertainty	Standard Uncertainty
Measurand	Individual and Spatial	$u_{m,ind}$	A	Normal	0.20 °C	0.10 °C	0.20 °C	0.10 °C
	Temporal	$u_{m,temp}$	A	Rectangular	0.25 °C	0.09 °C	0.50 °C	0.18 °C
	Environmental	$u_{m,\;env}$	B	Rectangular	0.25 °C	0.09 °C	0.50 °C	0.18 °C
Instrument	Calibration	$u_{i,cal}$	B	Normal	0.15 °C	0.08 °C	0.15 °C	0.08 °C
	Drift	$u_{i,drift}$	B	Normal	0.10 °C	0.05 °C	0.10 °C	0.05 °C
	Temperature resolution	$u_{i,res}$	A	Normal	0.10 °C	0.05 °C	0.10 °C	0.05 °C
	Response time	$u_{i,time}$	A/B	Normal	negligible	-	negligible	-
Environmental Influence quantities	Temperature effect ^1^	$u_{e,temp}$	B	Rectangular	0.10 °C	0.04 °C	0.20 °C	0.07 °C
	Skin emissivity ^2^	$u_{e,emi}$	B	Rectangular	0.05 °C	0.02 °C	0.10 °C	0.04 °C
	Mean radiant temperature ^3^	$u_{e,mrt}$	B	Rectangular	0.05 °C	0.02 °C	0.20 °C	0.07 °C
Operator	Target uniformity	$u_{o,target}$	B	Rectangular	0.05 °C	0.02 °C	0.05 °C	0.02 °C
	Angle incidence	$u_{o,angle}$	A/B	Normal	negligible	-	negligible	-
Composed Uncertainty		fixed threshold			0.40 °C	0.20 °C	0.62 °C	0.31 °C
		statistical threshold			0.28 °C	0.14 °C	0.42 °C	0.21 °C

^1^ Evaluated on the basis of a device temperature between 20 ± 2 °C (20 ± 5 °C) for indoor (outdoor) conditions. ^2^ Evaluated on the basis of a skin emissivity between 0.980 ± 0.003 (0.980 ± 0.006) for indoor (outdoor). ^3^ Evaluated on the basis of a mean radiant temperature between 20 ± 2 °C (20 ± 10 °C) for indoor (outdoor) conditions.

**Table 3:** Example of the proposed temperature screening protocol in indoor conditions.

First Step Noncontact Temperature (Forehead)	Second Step Contact Temperature (Axillary)	Action
t ≤ 35.5 °C	-	access allowed
35.5 < t ≤ 36.3 °C	t ≤ 37.3 °C	access allowed
	t > 37.3 °C	access denied
t > 36.3 °C	-	access denied

## Data Availability

Data sharing is not applicable to this article.
